# Effects of Dietary Nitrate Supplementation on Back Squat and Bench Press Performance: A Systematic Review and Meta-Analysis

**DOI:** 10.3390/nu15112493

**Published:** 2023-05-27

**Authors:** Rachel Tan, Adam Pennell, Sean T. Karl, Jordan K. Cass, Katherine Go, Tom Clifford, Stephen J. Bailey, Cooker Perkins Storm

**Affiliations:** 1 Department of Sports Medicine, Pepperdine University, Malibu, CA 90263, USA; adam.pennell@pepperdine.edu (A.P.); sean.karl@pepperdine.edu (S.T.K.); jordy.cass@pepperdine.edu (J.K.C.); katherine.go@pepperdine.edu (K.G.); cooker.storm@pepperdine.edu (C.P.S.); 2 School of Sport, Exercise, and Health Sciences, Loughborough University, Loughborough LE11 3TU, UK; t.clifford@lboro.ac.uk (T.C.); s.bailey2@lboro.ac.uk (S.J.B.)

**Keywords:** nitric oxide, beetroot, skeletal muscle, ergogenic aid, resistance exercise, weightlifting

## Abstract

This systematic review and meta-analysis investigated the influence of dietary nitrate supplementation on resistance exercise performance according to the PRISMA guidelines. Searches were conducted on MEDLINE, PubMed, ScienceDirect, Scopus and SPORTDiscus databases up to April 2023. Inclusion criteria were adult resistance-trained males who supplemented with a nitrate-rich supplement and nitrate-deficient placebo to assess repetitions-to-failure (RTF), peak power, mean power, peak velocity, and/or mean velocity during back squat and bench press exercise. A random effects model was performed on six studies and showed that nitrate supplementation improved RTF (standardized mean difference [SMD]: 0.43, 95% confidence intervals [95% CI]: 0.156 to 0.699, *p* = 0.002), mean power (SMD: 0.40, 95% CI: 0.127 to 0.678, *p* = 0.004), and mean velocity (SMD: 0.57, 95% CI: 0.07 to 1.061, *p* = 0.025) but had no effect on peak power (SMD: 0.204, 95% CI: −0.004 to 0.411, *p* = 0.054) or peak velocity (SMD: 0.00, 95% CI: −0.173 to 0.173, *p* = 1.000) when back squat and bench press were combined. Subgroup analyses revealed that back squats were more likely to be enhanced and that a dosing regimen may influence the efficacy of nitrate supplementation. Overall, nitrate supplementation had a small beneficial effect on some aspects of resistance exercise performance, but there were limited studies available and the variability was large. Additional studies that focus on upper and lower body resistance exercise and nitrate dosage are required to elucidate the efficacy of dietary nitrate supplementation on resistance exercise performance.

## 1. Introduction

In an attempt to enhance exercise performance, consumption of dietary supplements is a common practice employed by athletes of various competitive standards [1]. A recent consensus statement from the International Olympic Committee suggested that only five dietary supplements demonstrated a firm evidence basis for enhancing exercise performance [2]. Dietary nitrate (NO_3_^−^) is listed as one of these supplements and is also of appeal since it can be administered through natural food sources [3]. Dietary NO_3_^−^ supplementation is most commonly provided as a low (70 mL providing ~6.5 mmol of NO_3_^−^) to moderate dose (140 mL providing ~13 mmol of NO_3_^−^) of concentrated NO_3_^−^-rich beetroot juice [4]. While there are numerous systematic reviews and meta-analyses supporting a small ergogenic effect of potential dietary NO_3_^−^ supplementation, these have focused on performance outcomes during continuous submaximal cycling, as well as single and repeated bouts of high-intensity exercise [5,6,7,8,9]. Since dietary NO_3_^−^ is converted to nitrite (NO_2_^−^) with NO_2_^−^ subsequently reduced to nitric oxide (NO) [10] the ergogenic effects of dietary NO_3_^−^ supplementation have been attributed to its potential as a precursor for the multifaceted physiological signaling molecule, NO.

Successful execution of various sport-specific movements is dependent on effective power production by locomotor muscles, which is a product of muscle contractile force and velocity. Dietary NO_3_^−^ supplementation has been reported to improve muscle power output in humans, in various settings, as reflected by increases in knee extension strength and power output [11,12,13,14] and cycling peak power output [15,16,17]. Improved power output and performance have been observed at higher compared to lower contractile velocities after NO_3_^−^ supplementation in knee extensions [13] and cycling [18], with some evidence that NO_3_^−^ supplementation can also improve peak contractile velocity [13]. Therefore, improvements in muscle strength, contractile velocity, and power output after NO_3_^−^ supplementation may be expected to contribute to an increased number of reps-to-failure (RTF) at a given percentage of one-repetition maximum (%1RM) during resistance exercise.

The physiological mechanisms that may contribute to improved contractile velocity, power output, and RTF after NO_3_^−^ supplementation include a lower energy cost of force production [19,20], improved excitation–contraction coupling [21,22,23,24], and more pronounced physiological augmentations to calcium handling and blood flow in type II muscle fibers [24,25]. Furthermore, NO_3_^−^ supplementation may improve RTF and intermittent-type exercise by enhancing recovery between repetitions and sets owing to more rapid phosphocreatine resynthesis rates [26], attenuating accumulation of fatigue-associated metabolites [19], and/or facilitating a more homogenous skeletal muscle blood flow distribution [25]. Therefore, dietary NO_3_^−^ supplementation has the potential to enhance exercise performance in short-duration and single and/or multiple-repetition exercise bouts requiring movements with a high degree of strength, power, and velocity, and thus the recruitment of type II muscle fibers, such as resistance exercise [27]. However, compared to the >100 studies conducted in cycling and running performance, few studies have examined the influence of dietary NO_3_^−^ supplementation on resistance exercise performance (e.g., muscle power, velocity, RTF). Thus, there remains no consensus as to whether NO_3_^−^ ingestion can improve resistance exercise performance. The purpose of this systematic review and meta-analysis was to examine the efficacy of dietary NO_3_^−^ supplementation on improving back squat and bench press performance in healthy adult males.

## 2. Materials and Methods

The protocol for the present systematic review and meta-analysis was preregistered on the Open Science Framework (OSF) database (osf.io/h4j6q, accessed on on 18 January 2023) and is reported according to the Preferred Reporting Items for Systematic Reviews and Meta-Analysis (PRISMA) guidelines [28] and the PICOS (participants, interventions, comparators, outcomes, study design) criteria [29].

### 2.1. Search Strategy and Study Selection

The literature search was conducted on MEDLINE, PubMed, ScienceDirect, Scopus, and SPORTDiscus databases, and included all literature published before 1 April 2023. A combination of keywords and subject headings were used as search terms: (“nitrate” OR “beetroot”) AND (“male” OR “men” OR “human”) AND (“resistance” OR “training” OR “performance” OR “ergogenic” OR “exercise”) AND/OR (“power” OR “strength” OR “squat” OR “bench press”). The search results were downloaded into Zotero v.6. (Corporation for Digital Scholarship, Vienna, VI, USA) and imported into a systematic review screening software (Covidence, Melbourne, VIC, AUS). Three authors (S.T.K., J.K.C., K.G.) screened titles and abstracts to determine eligibility and remove duplicates, and any disagreements were resolved through consensus. Three authors (S.T.K., J.K.C., K.G.) independently read and reviewed the articles and further eliminated articles based on the inclusion criteria.

The primary outcome variables included one or more of the following outcome variables: peak power output (P_peak_), mean power output (P_mean_), peak velocity (V_peak_), mean velocity (V_mean_), all measured by linear transducer, and/or RTF (measured by number of repetitions). Inclusion criteria were applied based on the PICOS criteria (Supplementary Materials Table S1).

### 2.2. Quality Assessment

Risk of bias was assessed with the PEDro scale by two authors (J.K.C., K.G.) [30]. The PEDro scale criteria included 11 components: (1) specified eligibility criteria, (2) random allocation, (3) concealed allocation, (4) similar baseline characteristics for each condition, (5) blinding of participants, (6) blinding of researchers, (7) blinding of outcome assessors, (8) obtained key outcome for more than 85% of participants, (9) intention to treat, (10) between-group results reported for at least one key outcome, and (11) measures of variability and point measures provided for at least one key outcome. For each of the included studies, the PEDro criteria were categorized as poor (0–3), good (6–8), or excellent (9–10). Funnel plots and Egger’s regression tests were used to assess publication bias and were performed in Comprehensive Meta-Analysis version 4 software (Biostat Inc., Englewood, NJ, USA).

### 2.3. Data Extraction

A standardized data extraction sheet was developed on Microsoft Excel to extract study characteristics and performance outcomes. Three authors independently extracted study details (S.T.K., J.K.C., K.G.). A fourth author reviewed data extraction for accuracy and resolved any conflicts (R.T.). Data extracted included: participant characteristics (e.g., number, age, height, body mass, training status), supplementation regimen (dose, timing, frequency, and vehicle of administration), plasma [NO_3_^−^] and [NO_2_^−^], exercise protocol, exercise modality (free weights back squat, free weights bench press, Smith-machine bench press, Smith-machine back squat, fly-wheel back squat), exercise intensity as %1RM, and performance outcomes (P_peak_, P_mean_, V_peak_, V_mean_, RTF). Two authors (J.K.C., K.G.) completed data input of extracted data into the Comprehensive Meta-Analysis version 4 software (Biostat Inc., Englewood, NJ, USA) for statistical analysis. A third author verified the accuracy of the transferred data (S.T.K.). Mean and standard deviations were independently extracted by three authors (S.T.K., J.K.C., K.G.) and reviewed by a fourth author (R.T.). Group mean and standard deviation data were not presented in table or text for two studies and the authors were contacted [31,32]. Only one author responded and provided the missing mean and standard deviation data [31]. The means and standard deviations that were not available in the full-text publication [32] were extracted using an online software (WebPlotDigitizer, Version 4.3) by one author (K.G.) and verified by a second author (S.T.K.).

### 2.4. Meta-Analyses

Comprehensive Meta-Analysis version 4 software (Biostat Inc., Englewood, NJ, USA) was used for all analyses. Given the heterogeneity between studies (a priori significance was *p* < 0.05), a random-effects model was used to estimate the magnitude of effect of nitrate supplementation on performance variables. Hedges’ *g* effect sizes were calculated for each outcome; small, moderate, and large effects were defined as 0.20–0.49, 0.50–0.79, and ≥0.80, respectively [33]. Subgroup analyses was conducted for potential moderator variables of dose and type of exercise. The pooled data for each primary outcome variable and subgroup analyses are presented as standardized mean differences (SMD), 95% confidence intervals (95% CI) and forest plots. If individual studies had included multiple performance outcomes (e.g., P_peak_ in back squat and bench press), or used more than one dosing regimen (e.g., RTF after acute and multi-day NO_3_^−^ supplementation), SMDs were calculated for each of the performance variables measured within the study and were included in the same forest plot.

#### 2.4.1. Heterogeneity Assessment

Heterogeneity was assessed with Chi^2^ and *I*^2^ tests calculated in Comprehensive Meta-Analysis version 4 software (Biostat Inc., Englewood, NJ, USA) [34]. Values were defined as small (25–50%), medium (50–75%), and large (>75%) heterogeneity for *I*^2^, and significance was *p* ≤ 0.10 for Chi^2^ [34].

#### 2.4.2. Subgroup Analysis

Two subgroup analyses were performed on: (1) NO_3_^−^ dose (low: 6–7 mmol of NO_3_^−^ i.e., 1 × 70 mL NO_3_^−^-rich beetroot shots [Beet It; James Whyte Drinks; UK] vs. moderate: 12–13 mmol of NO_3_^−^ i.e., 2 × 70 mL NO_3_^−^-rich beetroot shots [Beet It; James Whyte Drinks; UK]) and (2) type of exercise (back squat vs. bench press).

## 3. Results

### 3.1. Study Selection

**Figure 1:**
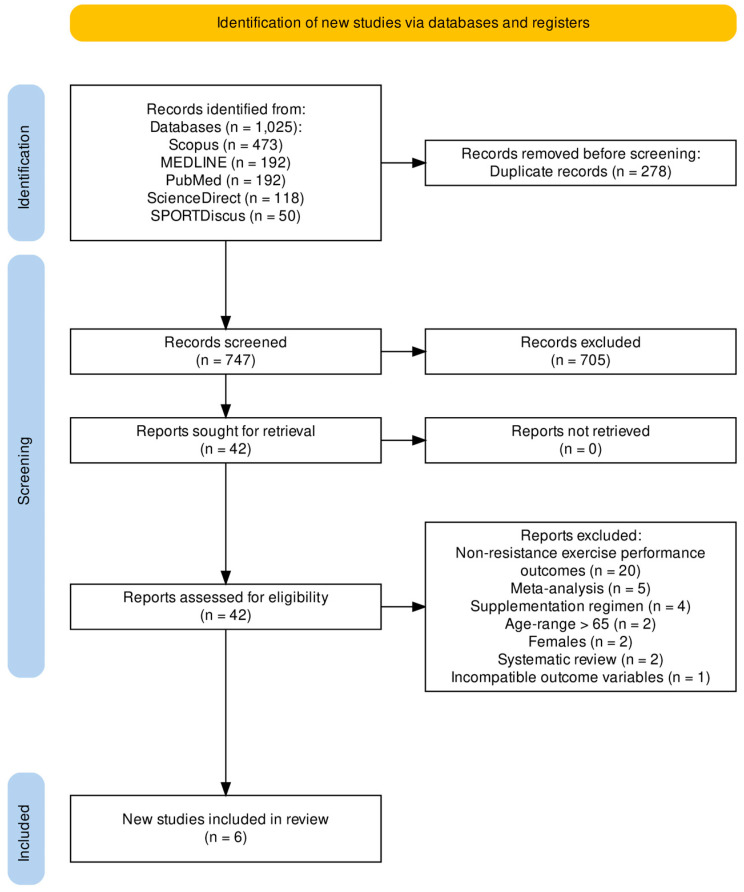
Literature search strategy.

The original search yielded a total of 1025 results. After the elimination of duplicates and performing title and abstract screening, 42 full-text articles were eligible for review. A total of six studies met the eligibility criteria for the present systematic review and meta-analysis (Figure 1).

### 3.2. Study Characteristics

A summary table of the six included studies is provided (Supplementary Materials Table S2).

In the six included studies, the sample size had a range of 11 to 18 participants and included a total of 92 participants aged 21 to 29 years. Participants were reported as resistance trained using various standards: (1) performed resistance exercise ≥2 times per week [35,36]; (2) ≥2 years of CrossFit and resistance exercise training experience with a back squat 1RM greater than 120 kg [31]; (3) ≥3 years of resistance exercise experience and a training frequency of ≥3 times and 3 h per week [32]; (4) moderately physically active 6 months prior to the study [37]; or (5) amateur sports players who participated a minimum of three times per week 1 year prior to the study [38]. Participant data for calculating BMI were only available for three studies and resulted in a range of 23.7 to 25.9 kg·m^−2^ [35,37,38]. The main supplementation method administered was NO_3_^−^-rich beetroot juice (BR; Beet It, Heart Beet Ltd; James White Drinks; Ipswich, UK) containing ~6 mmol of NO_3_^−^ in six studies [31,32,35,36,37,38]. Out of these six studies, only one study administered a NO_3_^−^-depleted beetroot juice as the placebo control where the taste, smell, and appearance were identical to BR (PL; Beet It, Heart Beet Ltd; James White Drinks; Ipswich, UK) [35]; three studies administered blackcurrant juice [32,36,37]; one study administered beetroot powder mixed in mineral water [31]; and one study did not specify the type of NO_3_^−^-depleted juice that was administered [38].

Four studies measured performance during back squats [31,35,37,38] whilst four studies measured performance during bench press [32,35,36,37]. For back squats, the modalities included back squats using free weights [31,35], a Smith machine [37], or a flywheel device [38]. Within bench press exercise, the modalities included bench press using free weights [35,36] or a Smith machine [32,37].

Five studies administered acute NO_3_^−^ supplementation as BR ranging from 2 h to 2.5 h prior to exercise [31,35,36,37,38]. Two studies provided an acute low NO_3_^−^ dose (~6.4 mmol of NO_3_^−^) [36,37] whilst three studies provided an acute moderate NO_3_^−^ dose (~13 mmol of NO_3_^−^) [31,35,38]. Two studies administered short-term (≥3 consecutive days) NO_3_^−^ supplementation including four days [35] and six days [32]. Out of the two studies, one study administered acute and short-term NO_3_^−^ supplementation from 2.5 h prior to exercise and four consecutive days of a moderate NO_3_^−^ dose (~6 mmol of NO_3_^−^ per day) [35], whilst one study administered six days of a low NO_3_^−^ dose (~6.4 mmol of NO_3_^−^ per day) but did not report the timing of ingestion [32].

One study included indices of NO bioavailability and measured plasma [NO_3_^−^] and [NO_2_^−^] via the gold standard method of gas phase chemiluminescence [35].

### 3.3. Risk of Bias

There were no studies excluded based on the PEDro scale (Supplementary Materials Table S3).

### 3.4. Publication Bias

Although it is conventional to require ten or more studies to reach adequate statistical power for funnel plots [39], these were still calculated for all primary variables of interest except for P_peak_, as there were too few studies for the software to compute a funnel plot (*n* = 2). As none of the variables had ten or more studies, all funnel plots were interpreted with caution and viewed as descriptive (as opposed to inferential) heuristics. Funnel plot figures are displayed in the Supplementary Materials (Figures S1–S4) for visual inspection of potential publication bias. Although no consistent evidence of publication bias was found, one study [38] was identified as potentially having a publication bias based on visual inspection on the funnel plot.

### 3.5. Meta-Analysis

#### 3.5.1. Repetitions-to-Failure

Data for RTF are displayed in the Supplementary Materials (Figure S5). Five studies measured RTF [31,32,35,36,37]. RTF was significantly improved following dietary NO_3_^−^ supplementation (SMD: 0.427, 95% CI: 0.156 to 0.688, *p* = 0.002, *n* = 5) and there was significant heterogeneity among these studies (Chi^2^ = 14.94; *I*^2^ = 46%; *p* = 0.06).

#### 3.5.2. Peak Power Output

Data for P_peak_ are displayed in the Supplementary Materials (Figure S6). Four studies measured P_peak_ [35,36,37,38]. There was no significance difference in P_peak_ following dietary NO_3_^−^ supplementation (SMD: 0.204, 95% CI: −0.004 to 0.411, *p* = 0.05, *n* = 4) and there was significant heterogeneity (Chi^2^ = 26.57; *I*^2^ = 51%; *p* = 0.01).

#### 3.5.3. Mean Power Output

Data for P_mean_ are displayed in the Supplementary Materials (Figure S7). Three studies measured P_mean_ [35,36,38]. P_mean_ was significantly improved following dietary NO_3_^−^ supplementation (SMD: 0.403, 95% CI: 0.127 to 0.678, *p* = 0.004, *n* = 3) and there was significant heterogeneity (Chi^2^ = 18.85; *I*^2^ = 58%; *p* = 0.016).

#### 3.5.4. Peak Velocity

Data for V_peak_ are displayed in the Supplementary materials (Figure S8). Two studies measured V_peak_ [35,37]. There was no significance difference in V_peak_ following dietary NO_3_^−^ supplementation (SMD: 0.000, 95% CI: −0.173 to 0.173, *p* = 1.00, *n* = 2) and the heterogeneity could not be computed due to the low number of studies.

#### 3.5.5. Mean Velocity

Data for V_mean_ are displayed in the Supplementary materials (Figure S9). Two studies measured V_mean_ [35,36]. V_mean_ was significantly improved following dietary NO_3_^−^ supplementation (SMD: 0.565, 95% CI: 0.07 to 1.061, *p* = 0.025, *n* = 2) and there was significant heterogeneity (Chi^2^ = 13.48; *I*^2^ = 70%; *p* = 0.009).

#### 3.5.6. Subgroup Analyses: RTF

Data for subgroup analyses by dose for RTF are displayed in the Supplementary Materials (Figure S10). RTF was significantly improved following dietary NO_3_^−^ supplementation provided as 6.4 mmol of NO_3_^−^ (SMD: 0.745, 95% CI: 0.417 to 1.073, *p* < 0.0001, *n* = 3) but not when provided as 13 mmol of NO_3_^−^ (SMD: 0.187, 95% CI: −0.081 to 0.456, *p* = 0.172, *n* = 2).

Data for subgroup analyses by exercise modality for RTF are displayed in the Supplementary Materials (Figure S11). RTF was significantly improved following dietary NO_3_^−^ supplementation during back squats (SMD: 0.416, 95% CI: 0.011 to 0.822, *p* = 0.044, *n* = 3) and bench press (SMD: 0.449, 95% CI: 0.038 to 0.861, *p* = 0.032, *n* = 4).

#### 3.5.7. Subgroup Analyses: Peak Power Output

Data for subgroup analyses by dose for P_peak_ are displayed in the Supplementary Materials (Figure S12). P_peak_ was significantly improved following dietary NO_3_^−^ supplementation provided as 13 mmol of NO_3_^−^ (SMD: 0.368, 95% CI: 0.062 to 0.674, *p* = 0.018, *n* = 2) but not when provided as 6.4 mmol of NO_3_^−^ (SMD: −0.031, 95% CI: −0.263 to 0.200, *p* = 0.790, *n* = 1).

Data for subgroup analyses by exercise modality for P_peak_ are displayed in the Supplementary Materials (Figure S13). P_peak_ was significantly improved following dietary NO_3_^−^ supplementation during back squats (SMD: 0.356, 95% CI: 0.087 to 0.624, *p* = 0.009, *n* = 3) but not bench press (SMD: −0.076, 95% CI: −0.323 to 0.170, *p* = 0.542, *n* = 2).

#### 3.5.8. Subgroup Analyses: Mean Power Output

Data for subgroup analyses by dose for P_mean_ are displayed in the Supplementary Materials (Figure S14). P_mean_ was significantly improved following dietary NO_3_^−^ supplementation provided as 13 mmol of NO_3_^−^ (SMD: 0.359, 95% CI: 0.071 to 0.647, *p* = 0.015, *n* = 2) and provided as 6.4 mmol of NO_3_^−^ (SMD: 0.860, 95% CI: 0.168 to 1.522, *p* = 0.015, *n* = 1).

Data for subgroup analyses by exercise modality for P_mean_ are displayed in the Supplementary Materials (Figure S15). P_mean_ was significantly improved following dietary NO_3_^−^ supplementation during back squats (SMD: 0.500, 95% CI: 0.193 to 0.806, *p* = 0.001, *n* = 2) but not bench press (SMD: 0.200, 95% CI: −0.342 to 0.742, *p* = 0.469, *n* = 2).

#### 3.5.9. Subgroup Analyses: Peak Velocity

There were no statistical differences in subgroup analyses in V_peak_ for dose or exercise modality (Supplementary Materials, Figures S16 and S17).

### 3.6. Subgroup Analyses: Mean Velocity

Data for subgroup analyses by dose for V_peak_ are displayed in the Supplementary Materials (Figure S18). V_peak_ was significantly improved following dietary NO_3_^−^ supplementation provided as 6.4 mmol of NO_3_^−^ (SMD: 0.670, 95% CI: 0.276 to 1.724, *p* = 0.003, *n* = 1) but not when provided as 13 mmol of NO_3_^−^ (SMD: 0.473, 95% CI: −0.087 to 1.032, *p* = 0.098, *n* = 1).

Data for subgroup analyses by exercise modality for V_mean_ are displayed in the Supplementary Materials (Figure S19). V_mean_ was significantly improved following dietary NO_3_^−^ supplementation during back squats (SMD: 1.000, 95% CI: 0.546 to 1.454, *p* < 0.0001, *n* = 1) but not bench press (SMD: 0.282, 95% CI: −0.292 to 0.857, *p* = 0.335, *n* = 2).

## 4. Discussion

This is the first systematic review and meta-analysis to have examined the potential ergogenic effects of dietary NO_3_^−^ supplementation on resistance exercise performance. The main novel findings were that dietary NO_3_^−^ supplementation had a small effect on improving RTF, P_mean_, and V_mean_ during resistance exercise, but that there were no significant improvements to P_peak_ or V_peak_. However, given the limited number of included studies, these findings should be interpreted cautiously. Indeed, out of the six included studies, there were five, three, two, two, and two studies that measured RTF, P_peak_, P_mean_, V_peak_, and V_mean_, respectively. Moreover, all of the studies had small sample sizes and wide confidence intervals. Therefore, while preliminary evidence is encouraging, the findings of this meta-analysis underscore the need for further research to evaluate the efficacy of NO_3_^−^ supplementation to improve aspects of resistance exercise performance.

Most studies examining the ergogenic potential of dietary NO_3_^−^ supplementation have been conducted using cycling or running as the exercise modality, with limited attention directed towards assessing its ergogenic potential during resistance exercise. Nonetheless, the findings from our meta-analysis in resistance exercise aligns with previous meta-analyses on running and cycling performance, all of which reported that dietary NO_3_^−^ supplementation confers performance enhancement with a small effect [8,40,41]. However, the findings from the current meta-analysis should be interpreted cautiously given the small effect sizes and wide confidence intervals observed for all of the performance outcome. These observations may be underpinned by interstudy variability between studies, a limited number of available studies, and the small sample sizes of the included studies.

We found that within each performance outcome, there were studies that yielded extremely large effects whilst other studies had negligible or trivial effects. For example, in P_peak_ and P_mean_, Rodríguez-Fernandez et al. [38] had large SMDs concomitant with upper limit confidence intervals that were very large effects of NO_3_^−^ during back squats. In contrast, other studies had negligible or trivial SMDs concomitant with small to moderate upper limits [35,37] which highlights the discrepancy in the effect sizes between studies. Similarly, although the overall pooled estimate of effect size for NO_3_^−^ on RTF was small, the wide confidence intervals are indicative of several studies which demonstrated large effects [31,32,36,37] while others have reported negligible to trivial effects [35]. Collectively, the variability in effect sizes between studies highlights the importance of interpreting results via quantitative synthesis of literature rather than from individual studies for insight into the potential true effect of NO_3_^−^ supplementation.

Variability could be due to the study design of resistance exercise protocols (e.g., number of sets, number of repetitions, rest periods, type of resistance exercise, exercise intensity, exercise protocol, phase of contraction), the performance outcomes (e.g., RTF, P_peak_, P_mean_, V_peak_, V_mean_), the reliability and validity of methods, whether confounding variables were controlled for (e.g., standardizing the tempo of the lift, technique accuracy, depth of movements, coaching techniques, dietary habits, supplementation intake, placebo-control, mood, training status, verbal encouragement, sleep, time of day), and interindividual physiological differences between participants which influence their “responsiveness” to dietary NO_3_^−^ supplementation, such as factors that influence NO bioavailability (see review: [42]). Therefore, despite the current meta-analysis suggesting that NO_3_^−^ supplementation can improve resistance exercise performance with a small effect size, there is still ambiguity surrounding the true effect size because of the divergent study designs and results between studies. Further high-quality investigations are required to elucidate the magnitude by which NO_3_^−^ supplementation improves resistance exercise performance.

The ergogenic effects of dietary NO_3_^−^ supplementation have been attributed to NO-mediated effects on type II muscle fibers [24,25], which are recruited during high power and high velocity contractions [43]. Since resistance exercise encompasses lower and upper body exercise protocols, and that various upper body muscle groups are comprised of a greater proportion of type II muscle fibers [44], it was suggested that the efficacy of NO_3_^−^ supplementation could be dependent on whether resistance exercise is being performed by the lower or upper body musculature [5]. The present meta-analysis found that only two studies directly compared back squats (lower body) and bench press (upper body) exercises [35,37], whilst the others focused on back squats [31,38] or bench press [32,36] independently. Subgroup analyses revealed that NO_3_^−^ supplementation had a similarly small beneficial effect on RTF in back squats and bench press. Furthermore, there were moderate, moderate and large effects of NO_3_^−^ on P_peak_, P_mean_, and V_mean_, respectively, in back squats only, although these results may be skewed by the large effects from a single study [38] as previously mentioned. It is important to acknowledge that the wide confidence intervals and limited number of studies make it difficult to interpret these findings. Notwithstanding, it is interesting to note that there was no effect of NO_3_^−^ supplementation on bench press performance, which contrasts with findings reporting benefits of NO_3_^−^ in sports that rely heavily on upper body strength like kayaking [45] and rowing [46]. It is possible that the efficacy of NO_3_^−^ supplementation is not solely determined by the fiber type composition of musculature but is, instead, dependent on the interplay between the rate of force development and exercise intensity, both of which would impact the activation of type II muscle fibers [43]. Clearly, more studies are needed to elucidate whether lower or upper body resistance exercise is more receptive to an ergogenic effect after NO_3_^−^ supplementation.

The current dosing recommendations indicate that a minimum effective dose of 5–9 mmol of NO_3_^−^ should significantly increase indices of NO bioavailability for eliciting performance enhancing effects [47]. In the present meta-analysis, all six of the included studies administered dietary NO_3_^−^ supplementation as NO_3_^−^-rich beetroot juice; however, only one study measured and reported plasma [NO_3_^−^] and [NO_2_^−^] following NO_3_^−^ supplementation to indicate the successful absorption and metabolism of dietary NO_3_^−^ [35]. Furthermore, only one study analyzed [NO_3_^−^] in the supplements to verify the dose provided in the NO_3_^−^ and placebo conditions [35], which is a limitation in the majority of the included studies, and an important measure to include given that a minimum effective dose is required to elicit beneficial effects [47]. Thus, the quality of the available evidence could be improved by including measures of plasma [NO_3_^−^] and [NO_2_^−^] to assess how NO_3_^−^ metabolism impacts the ergogenic potential of NO_3_^−^ on resistance exercise performance. Indeed, although the magnitude of elevation in plasma [NO_2_^−^] following NO_3_^−^ supplementation has been associated with the magnitude of performance enhancements in cycling [48,49] and neuromuscular strength [11], more studies are required to understand whether this notion holds true for different aspects of resistance exercise performance. Moreover, whilst current recommendations suggest that acute ingestion of 5–9 mmol of NO_3_^−^ would be sufficient to appreciably increase NO bioavailability [47], it is currently unknown how low (~6.4 mmol; 1 beetroot shot), moderate (~13 mmol; 2 beetroot shots), and high (≥26 mmol; ≥4 beetroot shots) doses impact the efficacy of NO_3_^−^ supplementation to improve resistance exercise performance since no dose-response studies have been conducted in this exercise modality. Although we ran subgroup analyses based on the NO_3_^−^ dose administered, we could only compare RTF following ~6.4 mmol (*n* = 3) and ~13 mmol (*n* = 2) given that the other performance outcomes had an insufficient number of studies. There was a significant trivial effect of NO_3_^−^ supplementation on RTF when administered as ~6.4 mmol but not when administered as ~13 mmol. It is possible that the wide confidence interval (95% CI: −0.08 to 0.46) and low number of studies for 13 mmol obscured any effects and further research is needed to determine the role of NO_3_^−^ dosage on resistance exercise performance. Whilst previous dose-response data indicated that increased NO_3_^−^ doses could induce more pronounced effects on exercise economy during cycling, at least up to 16 mmol of NO_3_^−^ [4], whether a dose-response effect occurs in performance enhancement is less clear. Indeed, it is possible that increasing NO_3_^−^ doses up to and beyond 16 mmol of NO_3_^−^ could elicit greater performance enhancements [49], or have no effect [50,51] or diminish effects [52], and as such, further research is required to assess the dose-response relationship between dietary NO_3_^−^ dose and resistance exercise performance. Taken together, this preliminary evidence suggests that ingestion with ≥6 mmol of NO_3_^−^ has potential to enhance resistance exercise performance but more work is required to elucidate how factors such as dose and type of exercise may impact the efficacy of NO_3_^−^ on resistance exercise performance.

To improve the quality of evidence, future studies are recommended to administer NO_3_^−^-depleted beetroot juice in the placebo condition to confidently isolate whether potential beneficial effects are a consequence of elevated NO bioavailability following NO_3_^−^ supplementation. Indeed, one study provided NO_3_^-^-depleted beetroot juice for the placebo condition [35] whilst the other studies provided blackcurrant juice [32,36,37], beetroot powder mixed in mineral water [31], or did not specify for the placebo condition [38] for the placebo condition, which may obscure the true effects of NO_3_^−^. Furthermore, future studies are recommended to standardize the classification of resistance-trained individuals for participant recruitment since differences in the training status of the participants in the included studies may have contributed to variability in the meta-analysis. For example, the participant population ranged from moderately physically active six months prior to the study [37], to amateur team sport players [38], to individuals who had performed resistance exercise ≥3 times per week for ≥3 years [32].

An important limitation of this systematic review and meta-analysis is the limited studies eligible for inclusion (*n* = 6), each of which had a small sample size. In addition, we included studies that measured multiple outcomes for the same participants, meaning they were double counted in the analysis. This was to increase our statistical power, and while this method was previously used in a meta-analysis examining the effect of NO_3_^−^ on exercise performance [8], we acknowledge that including multiple outcomes from individual studies may bias our findings towards those studies; therefore, these results should be interpreted cautiously. Furthermore, another limitation is that we were unable to investigate sex differences given that only one study had exclusively included female participants to investigate nitrate supplementation on resistance exercise performance at the time of the meta-analysis [53]. Given these key limitations, the results should be treated as preliminary, pending further studies.

## 5. Conclusions

Dietary NO_3_^−^ supplementation may induce small improvements to muscular endurance, power output, and velocity during back squats and bench press exercise. However, given the limited available data and significant heterogeneity between studies, further research is clearly needed to build upon these encouraging preliminary observations to evaluate the true ergogenic potential of NO_3_^−^ supplementation for resistance exercise.

## Data Availability

Data can be provided upon request.

## Supplementary Materials

The following supporting information can be downloaded at: https://www.mdpi.com/article/10.3390/nu15112493/s1, Figure S1. Funnel plot evaluating publication bias of trials assessing repetitions-to-failure (RTF) in groups following placebo and nitrate (n = 5, but 4 additional points are due to multiple outcomes from the same study if the study measured multiple primary performance outcomes); Figure S2. Funnel plot evaluating publication bias of trials assessing peak power output (P_peak_) in groups following placebo and nitrate (n = 3, but 11 additional points are due to multiple outcomes from the same study if the study measured multiple primary performance outcomes); Figure S3. Funnel plot evaluating publication bias of trials assessing mean power output (P_mean_) in groups following placebo and nitrate (n = 3, but 6 additional points are due to multiple outcomes from the same study if the study measured multiple primary performance outcomes); Figure S4. Funnel plot evaluating publication bias of trials assessing mean velocity (V_mean_) in groups following placebo and nitrate (n = 2, but 3 additional points are due to multiple outcomes from the same study if the study measured multiple primary performance outcomes). A funnel plot for peak velocity could not be computed owing to the low number of studies and available performance outcomes; Figure S5. Forest plot demonstrating repetitions-to-failure (RTF) in groups following placebo (A) and nitrate (B); Figure S6. Forest plot demonstrating peak power output (P_peak_) in groups receiving placebo (A) and nitrate (B); Figure S7. Forest plot demonstrating mean power output (P_mean_) in groups following placebo (A) and nitrate (B); Figure S8. Forest plot demonstrating peak velocity (V_peak_) in groups following placebo (A) and nitrate (B); Figure S9. Forest plot demonstrating mean velocity (V_mean_) in groups following placebo (A) and nitrate (B); Figure S10. Forest plot demonstrating subgroup analysis by dose for repetitions-to-failure (RTF) following placebo (A) and nitrate (B); Figure S11. Forest plot demonstrating subgroup analysis by exercise modality for repetitions-to-failure (RTF) following placebo (A) and nitrate (B); Figure S12. Forest plot demonstrating subgroup analysis by dose for peak power output (P_peak_) following placebo (A) and nitrate (B); Figure S13. Forest plot demonstrating subgroup analysis by exercise modality for peak power output (P_peak_) in groups receiving placebo (A) and nitrate (B); Figure S14. Forest plot demonstrating subgroup analysis by dose for mean power output (P_mean_) following placebo (A) and nitrate (B); Figure S15. Forest plot demonstrating subgroup analysis by exercise modality for mean power output (P_mean_) following placebo (A) and nitrate (B); Figure S16. Forest plot demonstrating subgroup analysis by dose for peak velocity (V_peak_) following placebo (A) and nitrate (B); Figure S17. Forest plot demonstrating subgroup analysis by exercise modality for peak velocity (V_peak_) following placebo (A) and nitrate (B); Figure S18. Forest plot demonstrating subgroup analysis by dose for mean velocity (V_mean_) following placebo (A) and nitrate (B); Figure S19. Forest plot demonstrating subgroup analysis by exercise modality for mean velocity (V_mean_) following placebo (A) and nitrate (B); Table S1: PICOS criteria; Table S2: Studies assessing the effects of dietary NO_3_^−^ supplementation on weightlifting performance in males; Table S3. Results from the quality assessment of studies based on the PEDro scale [30]. Total score for each study resulted from adding scores obtained for items 2–11.
