# Supplementary material for: Effects of Dietary Nitrate Supplementation on Back Squat and Bench Press Performance: A Systematic Review and Meta-Analysis
Source: Nutrients. 2023 May 27;15(11):2493. doi: 10.3390/nu15112493 (PMC10255616; doi:10.3390/nu15112493)
Supplement: Supplementary file 1 [file nutrients-15-02493-s001.zip › nutrients-2422333-supplementary.pdf]

Online Supplementary Material

Search Strategy for Medline

S1 ("nitrate" OR "beetroot") AND ("male" OR "men" OR "human") AND ("resistance" OR "training" OR "performance" OR "ergogenic" OR "exercise") AND ("power" OR "strength" OR "squat" OR "bench press") **(192)**

Search Strategy for PubMed

S2 ("nitrate" OR "beetroot") AND ("male" OR "men" OR "human") AND ("resistance" OR "training" OR "performance" OR "ergogenic" OR "exercise") AND ("power" OR "strength" OR "squat" OR "bench press") **(192)**

Search Strategy for ScienceDirect

S3 ("nitrate" OR "beetroot") AND ("resistance" OR "exercise" OR "performance" or "ergogenic") AND ("male" OR "men") AND ("power" OR "strength") **(118)**

Search Strategy for Scopus

S4 KEY(nitrate OR beetroot) AND (male OR men OR human) AND (resistance OR training OR performance OR ergogenic OR exercise) AND (power OR strength OR squat OR bench press) **(473)**

Search Strategy for SPORTDiscus

S5 ("nitrate" OR "beetroot") AND ("male" OR "men" OR "human") AND ("resistance" OR "training" OR "performance" OR "ergogenic" OR "exercise") AND/OR ("power" OR "strength" OR "squat" OR "bench press") **(50)**

Table S1. PICOS criteria

Parameter	Inclusion Criteria
Participant	Adult healthy resistance-trained human males, age 18-65 years
Intervention	NO_3^- supplementation, without other supplements ingested alongside the NO_3^- supplementation, and information was provided on the supplementation dose, timing, frequency, and vehicle of administration.
Comparator	Placebo with negligible NO_3^- content
Outcomes	Performance outcomes of power or velocity of contraction, or number of repetitions-to-failure (RTF) during resistance exercise.
Study Design	Randomized, double-blinded, crossover, placebo-controlled, trials. Only studies that were published in English and as original research (i.e., not a conference abstract or review) were included.

Table S2. Studies assessing the effects of dietary NO_3^- supplementation on weightlifting performance in males.

Author	Subjects	Supplementation	Exercise Protocol	Findings
Garnacho-Castaño et al. [31]	Eleven men<br><br>Age: 29 ± 4 y<br><br>Ht: 1.75 ± 0.06 m<br><br>Wt: 79 ± 5 kg	2.5-h min prior to exercise ingestion of 140 mL of NO_3^--rich BR (~13 mmol or ~800 mg of NO_3^-)	Wall ball shots and back squat: performed with Olympic barbell at 50% of 1RM. 2 sets x 90-s of wall balls + 60-s of back squat. Set 1: 90-s of wall balls plus 60-s of full back squat with 3 min recovery between exercises. Set 2: 90-s of wall balls plus 60-s of full back squat with no recovery between exercises.	↑ RTF: +13% (PL: 23 ± 4 vs. BR: 26 ± 2 reps)
Mosher et al. [32]	Twelve men<br><br>Age: 21 ± 2 y<br><br>Ht: 1.77 ± 0.04 m<br><br>Wt: 83 ± 10 kg	6 d of 1 × 70 mL NO_3^- rich BR supplementation (~6.4 mmol or ~400 mg of $NO_3^-{\cdot}d^{-1}$)	Smith machine bench press: 3 sets of RTF at 60%1RM with 2 min recovery between sets	↑ RTF: +19.4%
Ranchal-Sanchez et al. [37]	Twelve men<br><br>Age: 24 ± 3 y<br><br>Ht: 1.75 ± 0.08 m<br><br>Wt: 73 ± 9 kg	2-h prior to exercise ingestion of 1 × 70 mL NO_3^- rich BR (~6.4 mmol or ~400 mg of NO_3^-)	Smith machine bench press and back squat: 3 sets x RTF at 60−70−80%1RM with 2 min recovery between sets Following the eccentric phase of each rep, participants paused for 1.0−1.5-s	↔ P_{peak} back squat at 60, 70, and 80%1RM respectively (PL: 382 ± 111 vs. BR: 389 ± 117 W), (PL: 395 ± 107 vs. BR: 393 ± 116 W), and (PL: 378 ± 96 vs. BR: 377 ± 108 W)<br><br>↔ P_{peak} bench press at 60, 70, and 80%1RM respectively (PL: 292 ± 94 vs. BR: 289 ± 88 W), (PL: 238 ± 81 vs. BR: 242 ± 81 W), and (PL: 191 ± 55 vs. BR: 176 ± 66 W)<br><br>↔ V_{peak} back squat at 60, 70, and 80%1RM respectively (PL: 0.7 ± 0.1 vs. BR: 0.7 ± 0.1 $m{\cdot}s^{-1}$), (PL: 0.6 ± 0.1 vs. BR: 0.6 ± 0.1 $m{\cdot}s^{-1}$), and (PL: 0.5 ± 0.1 vs. BR: 0.5 ± 0.1 $m{\cdot}s^{-1}$)<br><br>↔ V_{peak} bench press at 60, 70, and 80%1RM respectively (PL: 0.6 ± 0.1 vs. BR: 0.6 ± 0.1 $m{\cdot}s^{-1}$), (PL: 0.4 ± 0.1 vs. BR: 0.4 ± 0.1 $m{\cdot}s^{-1}$), and (PL: 0.3 ± 0.1 vs. BR: 0.3 ± 0.1 $m{\cdot}s^{-1}$)<br><br>↑ RTF back squat: +23.4% (PL: 46 ± 16 vs. BR: 60 ± 20 reps)<br><br>↑ RTF total (sum bench press and back squat): +17.7% (PL: 75 ± 21 vs. BR: 89 ± 25 reps)
Rodríguez-Fernandez et al. [38]	Eighteen men<br><br>Age: 23 ± 5 y<br><br>Ht: 1.77 ± 0.10 m<br><br>Wt: 74 ± 10 kg	2.5-h prior to exercise ingestion of 140 mL NO_3^--rich BR (~13 mmol or ~800 mg of NO_3^-)	4 sets of half squats x 8 reps at inertial loads of 0.025, 0.050, 0.075, and 0.100 $kg{\cdot}m^2$ with 3 min recovery between sets	↑ P_{mean} for inertial loads of 0.025, 0.050, 0.075, and 0.100 respectively (PL: 1216 ± 278 vs. BR: 1434 ± 324 W), (PL: 1181 ± 295 vs. BR: 1396 ± 293 W), (PL: 1130 ± 259 vs. BR: 1382 ± 328 W), and (PL: 1033 ± 255 vs. BR: 1214 ± 273 W)<br><br>↑ P_{peak} for inertial loads of 0.025, 0.050, 0.075, and 0.100 respectively (PL: 2080 ± 376 vs. BR: 2447 ± 505 W), (PL: 2059 ± 334 vs. BR: 2351 ± 479 W), (PL 1940 ± 329 vs. BR: 2333 ± 492 W), and (PL: 1807 ± 347 vs. BR: 2078 ± 413 W)

Tan et al. [35]	Fourteen men<br><br>Age: 22 ± 5 y<br><br>Ht: 1.80 ± 0.06 m<br><br>Wt: 84 ± 17 kg	2.5-h, 4 d and 2.5 h prior to exercise ingestion of 2 x 70 mL NO_3^--rich BR (~5.9 mmol or ~400 mg of NO_3^- per 70 mL)	Back squat and bench press with barbell: warm-up x 3 reps, 5 reps at 40% 1RM, and 3 reps at 60% 1RM using 1-0-1-2 tempo interspersed with 2 min recovery between sets. 2 performance sets at 70% 1RM using 1-0-1-2 tempo interspersed with 2 min of rest. After 5 min recovery, 1 set x RTF at 60% 1RM performed without specified tempo	↔ P_{mean} during back squat after acute and chronic supplementation respectively (PL: 699 ± 163 vs. BR: 689 ± 162 W), (PL: 693 ± 189 vs. BR: 707 ± 162 W)<br><br>↔ P_{mean} during bench press after acute and chronic supplementation respectively (PL: 411 ± 107 vs. BR: 404 ± 90 W), (PL: 438 ± 124 vs. BR: 431 ± 114 W)<br><br>↔ P_{peak} during back squat after acute and chronic supplementation respectively (PL: 1700 ± 444 vs. BR: 1736 ± 461 W), (PL: 1810 ± 478 vs. BR: 1778 ± 461 W)<br><br>↔ P_{peak} during bench press after acute and chronic supplementation respectively (PL: 665 ± 179 vs. BR: 659 ± 163 W), (PL: 725 ± 216 vs. BR: 693 ± 183 W)<br><br>↔ V_{mean} during back squat after acute and chronic supplementation respectively (PL: 0.7 ± 0.1 vs. BR: 0.8 ± 0.1 $m{\cdot}s^{-1}$), (PL: 0.7 ±0.1 vs. BR: 0.8 ± 0.1 $m{\cdot}s^{-1}$)<br><br>↔ V_{mean} during bench press after acute and chronic supplementation respectively (PL: 0.6 ± 0.1 vs. BR: 0.6 ± 0.1 $m{\cdot}s^{-1}$), (PL: 0.6 ± 0.1 vs. BR: 0.6 ± 0.1 $m{\cdot}s^{-1}$)<br><br>↔ V_{peak} during back squat after acute and chronic supplementation respectively (PL: 1.5 ± 0.3 vs. BR: 1.5 ± 0.2 $m{\cdot}s^{-1}$), (PL: 1.5 ± 0.2 vs. BR: 1.5 ± 0.1 $m{\cdot}s^{-1}$)<br><br>↔ V_{peak} during bench press after acute and chronic supplementation respectively (PL: 0.9 ± 0.2 vs.BR: 0.9 ± 0.2 $m{\cdot}s^{-1}$), (PL: 0.9 ± 0.1 vs. BR: 0.9 ± 0.2 $m{\cdot}s^{-1}$)<br><br>↔ RTF back squat after acute and chronic supplementation respectively

				(PL: 28 ± 9 vs. BR: 28 ± 7 reps),<br>(PL: 29 ± 10 vs. BR: 30 ± 7 reps)<br><br>↑ RTF during bench press after acute and chronic supplementation respectively +5% (PL: 23 ± 4 vs. BR: 24 ± 5 reps),<br>(PL: 24 ± 4 vs. BR: 24 ± 5 reps)
Williams et al. [36]	Eleven men<br><br>Age: 22.1 ± 2.4 y<br><br>Ht: 1.71 ± 0.32 m<br><br>Wt: 89 ± 10 kg	2-h prior to exercise ingestion of 1 × 70 mL NO_3^- rich BR (~6.4 mmol or ~400 mg of NO_3^-)	Free-weight bench press: 2 sets x 2 explosive reps, 5 min recovery, then 3 sets x RTF at 70%1RM interspersed with 2 min recovery between sets	↑ P_{mean}: +19.5% (PL: 508 ± 118 vs. BR: 607 ± 112 W)<br><br>↑ V_{mean}: +6.5% (PL: 0.6 ± 0.1 vs. BR: 0.7 ± 0.1 $m \cdot s^{-1}$)<br><br>↑ RTF: +10.7% (PL: 28 ± 6 vs. BR: 31 ± 6 reps)

↑ = significant increase; ↓ = significant decrease; ↔ = no change; 1RM = one-repetition maximum; BR = beetroot juice; d = day; h = hours; Ht = height, kg = kilograms; m = meters; $m \cdot s^{-1}$ = meters per second; min = minutes; NO_3^- = nitrate; P_{mean} = mean power; P_{peak} = peak power; reps = repetitions; RTF = repetitions-to-failure; s = seconds; V_{mean} = mean velocity; V_{peak} = peak velocity; W = Watts; y = years; Wt: weight.

Table S3. Results from the quality assessment of studies based on the PEDro scale [30]. Total score for each study resulted from adding scores obtained for items 2-11.

Reference	Item 1	Item 2	Item 3	Item 4	Item 5	Item 6	Item 7	Item 8	Item 9	Item 10	Item 11	Total
Garnacho-Castaño et al. [31]	Y	Y	Y	Y	Y	Y	Y	Y	Y	Y	Y	10
Mosher et al. [32]	Y	Y	Y	Y	Y	Y	Y	Y	Y	Y	Y	10
Ranchal-Sanchez et al. [37]	Y	Y	Y	Y	Y	Y	Y	N	Y	Y	Y	9
Rodríguez-Fernandez et al. [38]	Y	Y	Y	Y	Y	Y	Y	N	Y	Y	Y	9
Tan et al. [35]	Y	Y	Y	Y	Y	Y	Y	Y	Y	Y	Y	10
Williams et al. [36]	Y	Y	Y	Y	Y	Y	Y	Y	Y	Y	Y	10

Y = yes; N = no

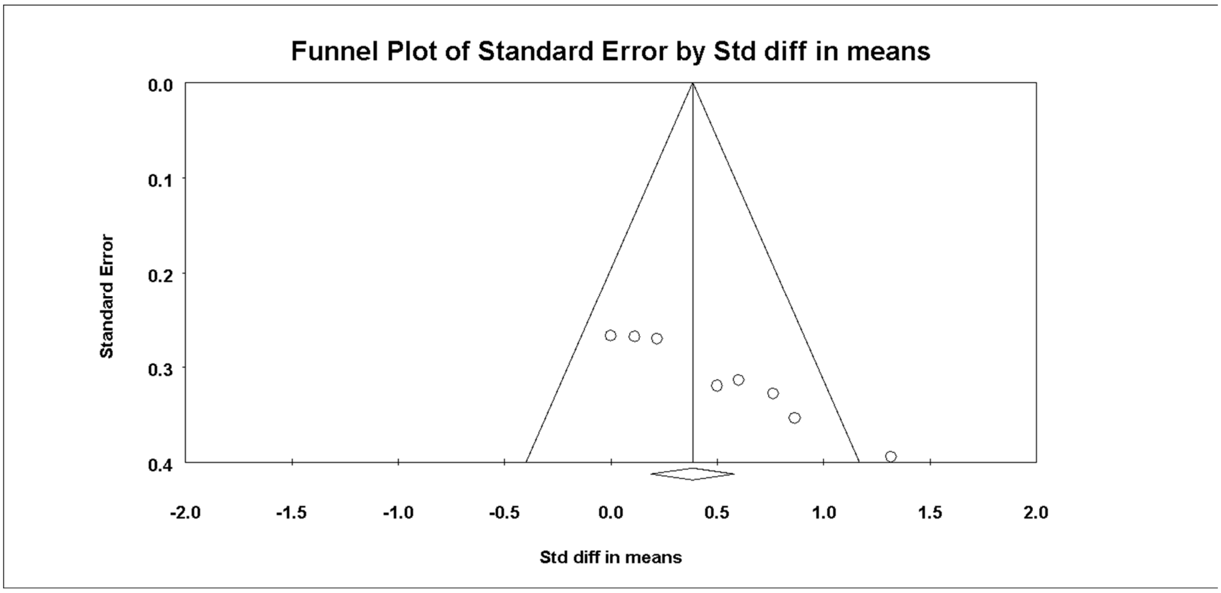

Figure S1. Funnel plot evaluating publication bias of trials assessing repetitions-to-failure (RTF) in groups following placebo and nitrate (n=5, but 4 additional points are due to multiple outcomes from the same study if the study measured multiple primary performance outcomes).

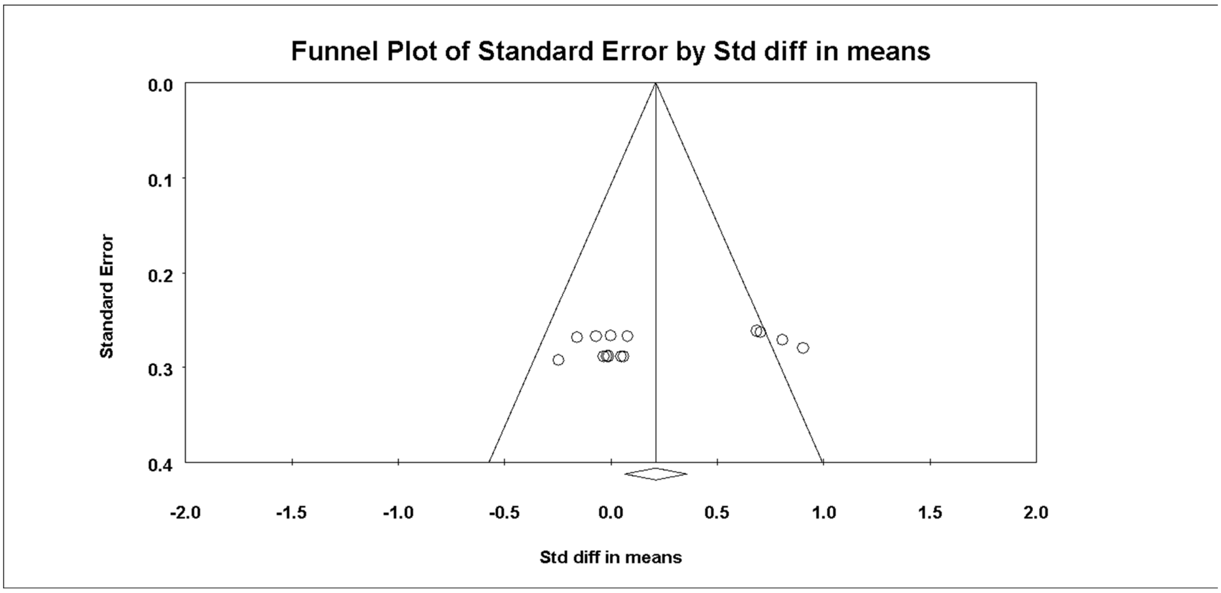

Figure S2. Funnel plot evaluating publication bias of trials assessing peak power output (P_{peak}) in groups following placebo and nitrate (n=3, but 11 additional points are due to multiple outcomes from the same study if the study measured multiple primary performance outcomes).

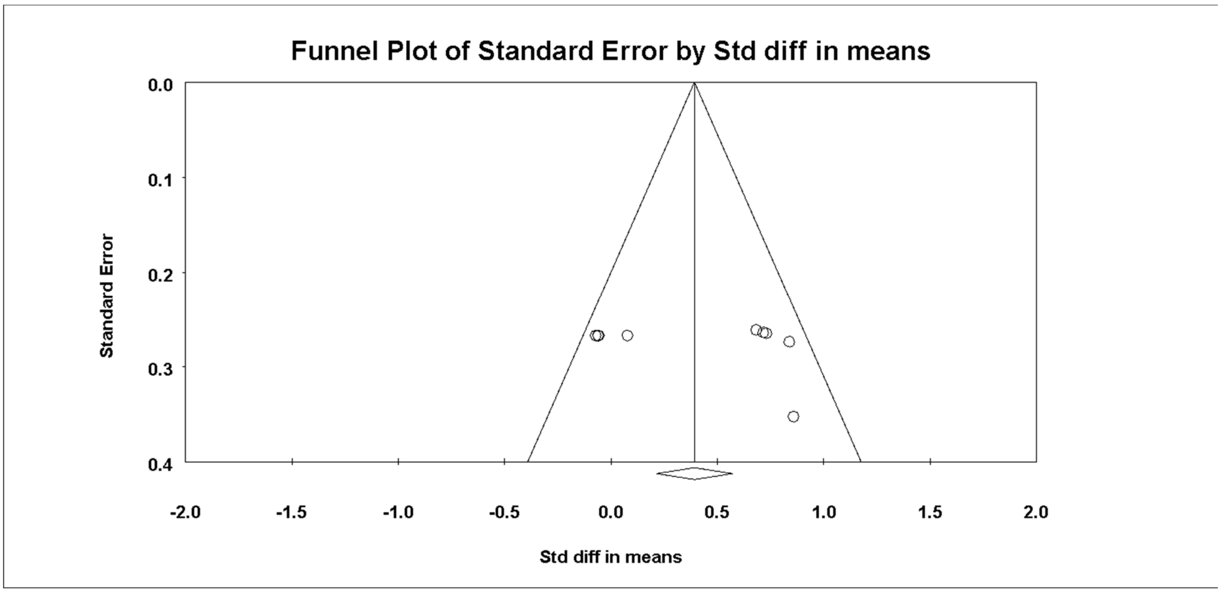

Figure S3. Funnel plot evaluating publication bias of trials assessing mean power output (P_{mean}) in groups following
placebo and nitrate (n=3, but 6 additional points are due to multiple outcomes from the same study if the study measured multiple primary performance outcomes).

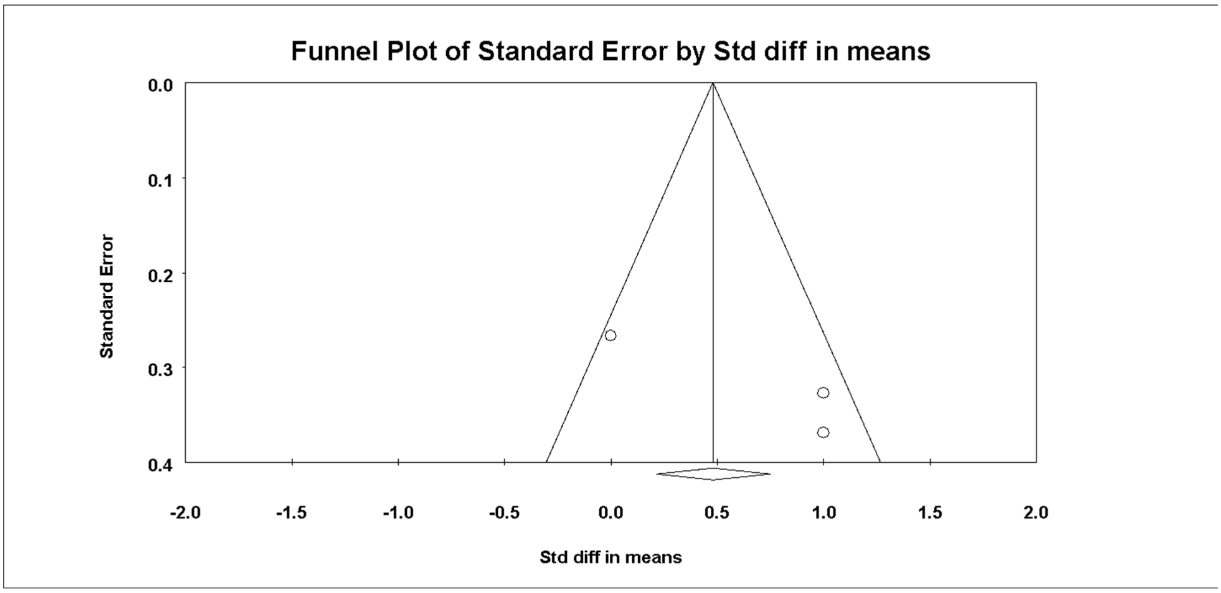

Figure S4. Funnel plot evaluating publication bias of trials assessing mean velocity (V_{mean}) in groups following placebo and nitrate (n=2, but 3 additional points are due to multiple outcomes from the same study if the study measured multiple primary performance outcomes). A funnel plot for peak velocity could not be computed owing to the low number of studies and available performance outcomes.

Meta Analysis

Study name	Subgroup within study	Std diff in means	Standard error	Variance	Lower limit	Upper limit	Z-Value	p-Value
Gamacho-Castano et al. (2022)	a	0.866	0.354	0.125	0.173	1.559	2.449	0.014
Ranchal-Sanchez et al. (2020)	a	0.764	0.328	0.108	0.121	1.407	2.328	0.020
Ranchal-Sanchez et al. (2020)	b	0.602	0.314	0.098	-0.013	1.217	1.919	0.055
Tan et al. (2022)	a	0.000	0.267	0.071	-0.524	0.524	0.000	1.000
Tan et al. (2022)	b	0.218	0.270	0.073	-0.312	0.748	0.807	0.420
Tan et al. (2022)	c	0.113	0.268	0.072	-0.413	0.638	0.420	0.675
Tan et al. (2022)	d	0.000	0.267	0.071	-0.524	0.524	0.000	1.000
Mosher et al. (2016)	a	1.317	0.395	0.156	0.544	2.091	3.339	0.001
Williams et al. (2020)	a	0.500	0.320	0.102	-0.127	1.127	1.563	0.118
Pooled		0.427	0.139	0.019	0.156	0.699	3.083	0.002
Prediction Interval		0.427			-0.315	1.169		

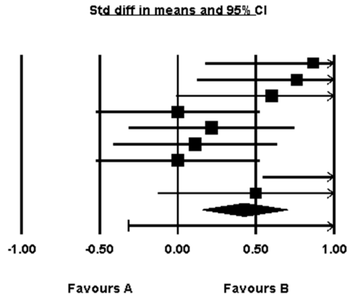

Meta Analysis

Figure S5. Forest plot demonstrating repetitions-to-failure (RTF) in groups following placebo (A) and nitrate (B).

Meta Analysis

Study name	Subgroup within study	Std diff in means	Standard error	Variance	Lower limit	Upper limit	Z-Value	p-Value
		Statistics for each study						
Randhal-Sanchez et al. (2020)	a	0.061	0.289	0.083	-0.505	0.628	0.212	0.832
Randhal-Sanchez et al. (2020)	b	-0.018	0.289	0.083	-0.584	0.548	-0.062	0.951
Randhal-Sanchez et al. (2020)	c	-0.010	0.289	0.083	-0.576	0.556	-0.034	0.973
Randhal-Sanchez et al. (2020)	d	-0.033	0.289	0.083	-0.599	0.533	-0.114	0.909
Randhal-Sanchez et al. (2020)	e	0.049	0.289	0.083	-0.517	0.616	0.171	0.864
Randhal-Sanchez et al. (2020)	f	-0.245	0.293	0.086	-0.819	0.329	-0.836	0.403
Rodriguez-Fernandez et al. (2021)	a	0.808	0.271	0.074	0.276	1.340	2.975	0.003
Rodriguez-Fernandez et al. (2021)	b	0.686	0.262	0.069	0.173	1.200	2.620	0.009
Rodriguez-Fernandez et al. (2021)	c	0.905	0.280	0.078	0.357	1.454	3.235	0.001
Rodriguez-Fernandez et al. (2021)	d	0.705	0.263	0.069	0.189	1.221	2.678	0.007
Tan et al. (2022)	a	0.080	0.268	0.072	-0.445	0.604	0.297	0.766
Tan et al. (2022)	b	-0.068	0.268	0.072	-0.593	0.456	-0.255	0.799
Tan et al. (2022)	c	0.000	0.267	0.071	-0.524	0.524	0.000	1.000
Tan et al. (2022)	d	-0.159	0.269	0.072	-0.686	0.368	-0.590	0.555
Pooled		0.204	0.106	0.011	-0.004	0.411	1.924	0.054
Prediction Interval		0.204			-0.455	0.862		

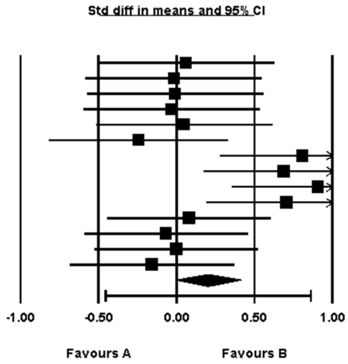

Meta Analysis

Figure S6. Forest plot demonstrating peak power output (P_{peak}) in groups receiving placebo (A) and nitrate (B).

Meta Analysis

Study name	Subgroup within study	Std diff in means	Standard error	Variance	Lower limit	Upper limit	Z-Value	p-Value
Rodriguez-Fernandez et al. (2021)	a	0.718	0.264	0.070	0.200	1.236	2.716	0.007
Rodriguez-Fernandez et al. (2021)	b	0.731	0.265	0.070	0.211	1.251	2.756	0.006
Rodriguez-Fernandez et al. (2021)	c	0.841	0.274	0.075	0.304	1.379	3.068	0.002
Rodriguez-Fernandez et al. (2021)	d	0.684	0.262	0.069	0.171	1.198	2.614	0.009
Tan et al. (2022)	a	-0.056	0.267	0.072	-0.581	0.468	-0.211	0.833
Tan et al. (2022)	b	0.079	0.268	0.072	-0.446	0.604	0.295	0.768
Tan et al. (2022)	c	-0.070	0.268	0.072	-0.595	0.454	-0.263	0.793
Tan et al. (2022)	d	-0.059	0.267	0.072	-0.583	0.466	-0.219	0.826
Williams et al. (2020)	a	0.860	0.353	0.125	0.168	1.552	2.437	0.015
Pooled		0.403	0.140	0.020	0.127	0.678	2.865	0.004
Prediction Interval		0.403			-0.421	1.227		

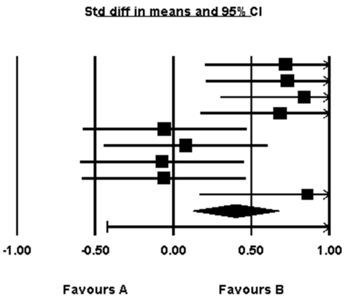

Meta Analysis

Figure S7. Forest plot demonstrating mean power output (P_{mean}) in groups following placebo (A) and nitrate (B).

Meta Analysis

Study name	Subgroup within study	Std diff in means	Standard error	Variance	Lower limit	Upper limit	Z-Value	p-Value
Ranchal-Sanchez et al. (2020)	a	0.000	0.289	0.083	-0.566	0.566	0.000	1.000
Ranchal-Sanchez et al. (2020)	b	0.000	0.289	0.083	-0.566	0.566	0.000	1.000
Ranchal-Sanchez et al. (2020)	c	0.000	0.289	0.083	-0.566	0.566	0.000	1.000
Ranchal-Sanchez et al. (2020)	d	0.000	0.289	0.083	-0.566	0.566	0.000	1.000
Ranchal-Sanchez et al. (2020)	e	0.000	0.289	0.083	-0.566	0.566	0.000	1.000
Ranchal-Sanchez et al. (2020)	f	0.000	0.289	0.083	-0.566	0.566	0.000	1.000
Tan et al. (2022)	a	0.000	0.267	0.071	-0.524	0.524	0.000	1.000
Tan et al. (2022)	b	0.000	0.267	0.071	-0.524	0.524	0.000	1.000
Tan et al. (2022)	c	0.000	0.267	0.071	-0.524	0.524	0.000	1.000
Tan et al. (2022)	d	0.000	0.267	0.071	-0.524	0.524	0.000	1.000
Pooled		0.000	0.088	0.008	-0.173	0.173	0.000	1.000
Prediction Interval								

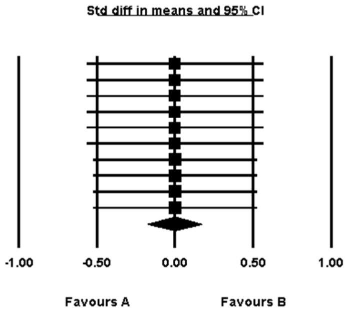

Meta Analysis

Figure S8. Forest plot demonstrating peak velocity (V_{peak}) in groups following placebo (A) and nitrate (B).

Meta Analysis

Study name	Subgroup within study	Std diff in means	Standard error	Variance	Lower limit	Upper limit	Z-Value	p-Value
Tan et al. (2022)	a	1.000	0.327	0.107	0.358	1.642	3.055	0.002
Tan et al. (2022)	b	1.000	0.327	0.107	0.358	1.642	3.055	0.002
Tan et al. (2022)	c	0.000	0.267	0.071	-0.524	0.524	0.000	1.000
Tan et al. (2022)	d	0.000	0.267	0.071	-0.524	0.524	0.000	1.000
Williams et al. (2020)	a	1.000	0.369	0.136	0.276	1.724	2.708	0.007
Pooled		0.565	0.253	0.064	0.070	1.061	2.236	0.025
Prediction Interval		0.565			-1.139	2.270		

Statistics for each study

Std diff in means and 95% CI

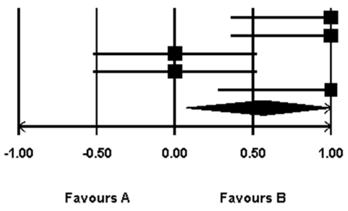

Meta Analysis

Figure S9. Forest plot demonstrating mean velocity (V_{mean}) in groups following placebo (A) and nitrate (B).

Meta Analysis

Group by Dose	Study name	Subgroup within study	Std diff in means	Standard error	Variance	Lower limit	Upper limit	Z-Value	p-Value
			Statistics for each study						
13 mmol	Gamacho-Castano et al. (2022)	a	0.866	0.354	0.125	0.173	1.559	2.449	0.014
13 mmol	Tan et al. (2022)	a	0.000	0.267	0.071	-0.524	0.524	0.000	1.000
13 mmol	Tan et al. (2022)	b	0.218	0.270	0.073	-0.312	0.748	0.807	0.420
13 mmol	Tan et al. (2022)	c	0.113	0.268	0.072	-0.413	0.638	0.420	0.675
13 mmol	Tan et al. (2022)	d	0.000	0.267	0.071	-0.524	0.524	0.000	1.000
13 mmol	Pooled		0.187	0.137	0.019	-0.081	0.456	1.366	0.172
13 mmol	Prediction Interval		0.187			-0.398	0.772		
6.4 mmol	Ranchal-Sanchez et al. (2020)	a	0.764	0.328	0.108	0.121	1.407	2.328	0.020
6.4 mmol	Ranchal-Sanchez et al. (2020)	b	0.602	0.314	0.098	-0.013	1.217	1.919	0.055
6.4 mmol	Mosher et al. (2016)	a	1.317	0.395	0.156	0.544	2.091	3.339	0.001
6.4 mmol	Williams et al. (2020)	a	0.500	0.320	0.102	-0.127	1.127	1.563	0.118
6.4 mmol	Pooled		0.745	0.167	0.028	0.417	1.073	4.449	0.000
6.4 mmol	Prediction Interval								
Overall	Pooled		0.411	0.106	0.011	0.203	0.619	3.876	0.000
Overall	Prediction Interval		0.411			-0.300	1.123		

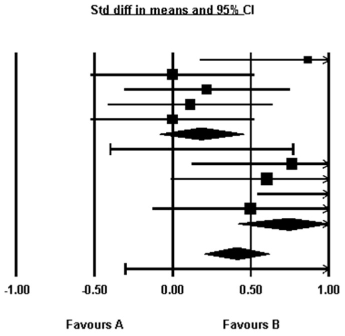

Meta Analysis

Figure S10. Forest plot demonstrating subgroup analysis by dose for repetitions-to-failure (RTF) following placebo (A) and nitrate (B).

Meta Analysis

Group by Exercise	Study name	Subgroup within study	Std diff in means	Standard error	Variance	Lower limit	Upper limit	Z-Value	p-Value
Bench Press	Ranchal-Sanchez et al. (2020)	b	0.602	0.314	0.098	-0.013	1.217	1.919	0.055
Bench Press	Tan et al. (2022)	c	0.113	0.268	0.072	-0.413	0.638	0.420	0.675
Bench Press	Tan et al. (2022)	d	0.000	0.267	0.071	-0.524	0.524	0.000	1.000
Bench Press	Mosher et al. (2016)	a	1.317	0.395	0.156	0.544	2.091	3.339	0.001
Bench Press	Williams et al. (2020)	a	0.500	0.320	0.102	-0.127	1.127	1.563	0.118
Bench Press	Pooled		0.449	0.210	0.044	0.038	0.861	2.140	0.032
Bench Press	Prediction Interval		0.449			-0.856	1.755		
Squat	Garnacho-Castano et al. (2022)	a	0.866	0.354	0.125	0.173	1.559	2.449	0.014
Squat	Ranchal-Sanchez et al. (2020)	a	0.764	0.328	0.108	0.121	1.407	2.328	0.020
Squat	Tan et al. (2022)	a	0.000	0.267	0.071	-0.524	0.524	0.000	1.000
Squat	Tan et al. (2022)	b	0.218	0.270	0.073	-0.312	0.748	0.807	0.420
Squat	Pooled		0.416	0.207	0.043	0.011	0.822	2.013	0.044
Squat	Prediction Interval		0.416			-1.089	1.922		
Overall	Pooled		0.433	0.147	0.022	0.144	0.721	2.936	0.003
Overall	Prediction Interval		0.433			-0.319	1.184		

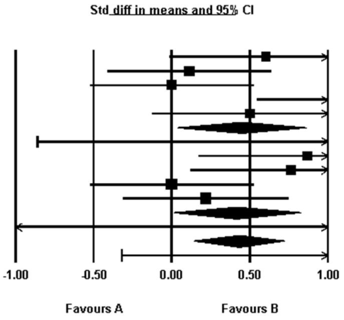

Meta Analysis

Figure S11. Forest plot demonstrating subgroup analysis by exercise modality for repetitions-to-failure (RTF) following placebo (A) and nitrate (B).

Meta Analysis

Group by Dose	Study name	Subgroup within study	Std diff in means	Standard error	Variance	Lower limit	Upper limit	Z-Value	p-Value
13.00	Rodriguez-Fernandez et al. (2021)	a	0.808	0.271	0.074	0.276	1.340	2.975	0.003
13.00	Rodriguez-Fernandez et al. (2021)	b	0.686	0.262	0.069	0.173	1.200	2.620	0.009
13.00	Rodriguez-Fernandez et al. (2021)	c	0.905	0.280	0.078	0.357	1.454	3.235	0.001
13.00	Rodriguez-Fernandez et al. (2021)	d	0.705	0.263	0.069	0.189	1.221	2.678	0.007
13.00	Tan et al. (2022)	a	0.080	0.268	0.072	-0.445	0.604	0.297	0.766
13.00	Tan et al. (2022)	b	-0.068	0.268	0.072	-0.593	0.456	-0.255	0.799
13.00	Tan et al. (2022)	c	0.000	0.267	0.071	-0.524	0.524	0.000	1.000
13.00	Tan et al. (2022)	d	-0.159	0.269	0.072	-0.686	0.368	-0.590	0.555
13.00	Pooled		0.368	0.156	0.024	0.062	0.674	2.356	0.018
13.00	Prediction Interval		0.368			-0.572	1.308		
6.40	Ranchal-Sanchez et al. (2020)	a	0.061	0.289	0.083	-0.505	0.628	0.212	0.832
6.40	Ranchal-Sanchez et al. (2020)	b	-0.018	0.289	0.083	-0.584	0.548	-0.062	0.951
6.40	Ranchal-Sanchez et al. (2020)	c	-0.010	0.289	0.083	-0.576	0.556	-0.034	0.973
6.40	Ranchal-Sanchez et al. (2020)	d	-0.033	0.289	0.083	-0.599	0.533	-0.114	0.909
6.40	Ranchal-Sanchez et al. (2020)	e	0.049	0.289	0.083	-0.517	0.616	0.171	0.864
6.40	Ranchal-Sanchez et al. (2020)	f	-0.245	0.293	0.086	-0.819	0.329	-0.836	0.403
6.40	Pooled		-0.031	0.118	0.014	-0.263	0.200	-0.266	0.790
6.40	Prediction Interval								
Overall	Pooled		0.114	0.094	0.009	-0.071	0.299	1.209	0.227
Overall	Prediction Interval		0.114			-0.536	0.764		

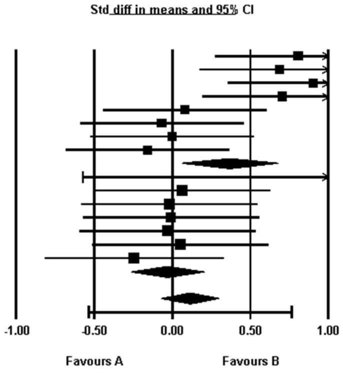

Meta Analysis

Figure S12. Forest plot demonstrating subgroup analysis by dose for peak power output (P_{peak}) following placebo (A) and nitrate (B).

Meta Analysis

Group by Exercise	Study name	Subgroup within study	Std diff in means	Standard error	Variance	Lower limit	Upper limit	Z-Value	p-Value
Bench Press	Randhal-Sanchez et al. (2020)	d	-0.033	0.289	0.083	-0.599	0.533	-0.114	0.909
Bench Press	Randhal-Sanchez et al. (2020)	e	0.049	0.289	0.083	-0.517	0.616	0.171	0.864
Bench Press	Randhal-Sanchez et al. (2020)	f	-0.245	0.293	0.086	-0.819	0.329	-0.836	0.403
Bench Press	Tan et al. (2022)	c	0.000	0.267	0.071	-0.524	0.524	0.000	1.000
Bench Press	Tan et al. (2022)	d	-0.159	0.269	0.072	-0.686	0.368	-0.590	0.555
Bench Press	Pooled		-0.076	0.126	0.016	-0.323	0.170	-0.609	0.542
Bench Press	Prediction Interval								
Squat	Randhal-Sanchez et al. (2020)	a	0.061	0.289	0.083	-0.505	0.628	0.212	0.832
Squat	Randhal-Sanchez et al. (2020)	b	-0.018	0.289	0.083	-0.584	0.548	-0.062	0.951
Squat	Randhal-Sanchez et al. (2020)	c	-0.010	0.289	0.083	-0.576	0.556	-0.034	0.973
Squat	Rodriguez-Fernandez et al. (2021)	a	0.808	0.271	0.074	0.276	1.340	2.975	0.003
Squat	Rodriguez-Fernandez et al. (2021)	b	0.686	0.262	0.069	0.173	1.200	2.620	0.009
Squat	Rodriguez-Fernandez et al. (2021)	c	0.905	0.280	0.078	0.357	1.454	3.235	0.001
Squat	Rodriguez-Fernandez et al. (2021)	d	0.705	0.263	0.069	0.189	1.221	2.678	0.007
Squat	Tan et al. (2022)	a	0.080	0.268	0.072	-0.445	0.604	0.297	0.766
Squat	Tan et al. (2022)	b	-0.068	0.268	0.072	-0.593	0.456	-0.255	0.799
Squat	Pooled		0.356	0.137	0.019	0.087	0.624	2.597	0.009
Squat	Prediction Interval		0.356			-0.435	1.147		
Overall	Pooled		0.121	0.093	0.009	-0.061	0.302	1.306	0.192
Overall	Prediction Interval		0.121			-0.528	0.769		

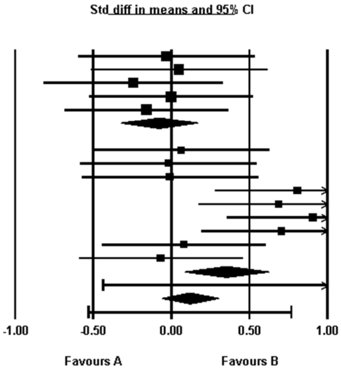

Meta Analysis

Figure S13. Forest plot demonstrating subgroup analysis by exercise modality for peak power output (P_{peak}) in groups receiving placebo (A) and nitrate (B).

Meta Analysis

Group by Dose	Study name	Subgroup within study	Std diff in means	Standard error	Variance	Lower limit	Upper limit	Z-Value	p-Value
13.00	Rodriguez-Fernandez et al. (2021)	a	0.718	0.264	0.070	0.200	1.236	2.716	0.007
13.00	Rodriguez-Fernandez et al. (2021)	b	0.731	0.265	0.070	0.211	1.251	2.756	0.006
13.00	Rodriguez-Fernandez et al. (2021)	c	0.841	0.274	0.075	0.304	1.379	3.068	0.002
13.00	Rodriguez-Fernandez et al. (2021)	d	0.684	0.262	0.069	0.171	1.198	2.614	0.009
13.00	Tan et al. (2022)	a	-0.056	0.267	0.072	-0.581	0.468	-0.211	0.833
13.00	Tan et al. (2022)	b	0.079	0.268	0.072	-0.446	0.604	0.295	0.768
13.00	Tan et al. (2022)	c	-0.070	0.268	0.072	-0.595	0.454	-0.263	0.793
13.00	Tan et al. (2022)	d	-0.059	0.267	0.072	-0.583	0.466	-0.219	0.826
13.00	Pooled		0.359	0.147	0.022	0.071	0.647	2.442	0.015
13.00	Prediction Interval		0.359			-0.500	1.217		
6.40	Williams et al. (2020)	a	0.860	0.353	0.125	0.168	1.552	2.437	0.015
6.40	Pooled		0.860	0.353	0.125	0.168	1.552	2.437	0.015
6.40	Prediction Interval								
Overall	Pooled		0.433	0.136	0.018	0.167	0.699	3.191	0.001
Overall	Prediction Interval		0.433			-0.387	1.252		

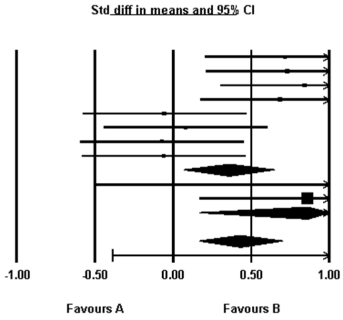

Meta Analysis

Figure S14. Forest plot demonstrating subgroup analysis by dose for mean power output (P_{mean}) following placebo (A) and nitrate (B).

Meta Analysis

Group by Exercise	Study name	Subgroup within study	Std diff in means	Standard error	Variance	Lower limit	Upper limit	Z-Value	p-Value
Bench Press	Tan et al. (2022)	c	-0.070	0.268	0.072	-0.595	0.454	-0.263	0.793
Bench Press	Tan et al. (2022)	d	-0.059	0.267	0.072	-0.583	0.466	-0.219	0.826
Bench Press	Williamset al. (2020)	a	0.860	0.353	0.125	0.168	1.552	2.437	0.015
Bench Press	Pooled		0.200	0.277	0.077	-0.342	0.742	0.723	0.469
Bench Press	Prediction Interval		0.200			-5.751	6.151		
Squat	Rodriguez-Fernandez et al. (2021)	a	0.718	0.264	0.070	0.200	1.236	2.716	0.007
Squat	Rodriguez-Fernandez et al. (2021)	b	0.731	0.265	0.070	0.211	1.251	2.756	0.006
Squat	Rodriguez-Fernandez et al. (2021)	c	0.841	0.274	0.075	0.304	1.379	3.068	0.002
Squat	Rodriguez-Fernandez et al. (2021)	d	0.684	0.262	0.069	0.171	1.198	2.614	0.009
Squat	Tan et al. (2022)	a	-0.056	0.267	0.072	-0.581	0.468	-0.211	0.833
Squat	Tan et al. (2022)	b	0.079	0.268	0.072	-0.446	0.604	0.295	0.768
Squat	Pooled		0.500	0.156	0.024	0.193	0.806	3.198	0.001
Squat	Prediction Interval		0.500			-0.377	1.376		
Overall	Pooled		0.427	0.136	0.019	0.161	0.694	3.141	0.002
Overall	Prediction Interval		0.427			-0.393	1.247		

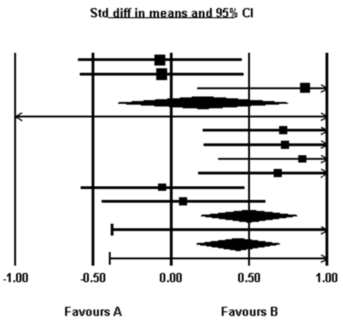

Meta Analysis

Figure S15. Forest plot demonstrating subgroup analysis by exercise modality for mean power output (P_{mean}) following placebo (A) and nitrate (B).

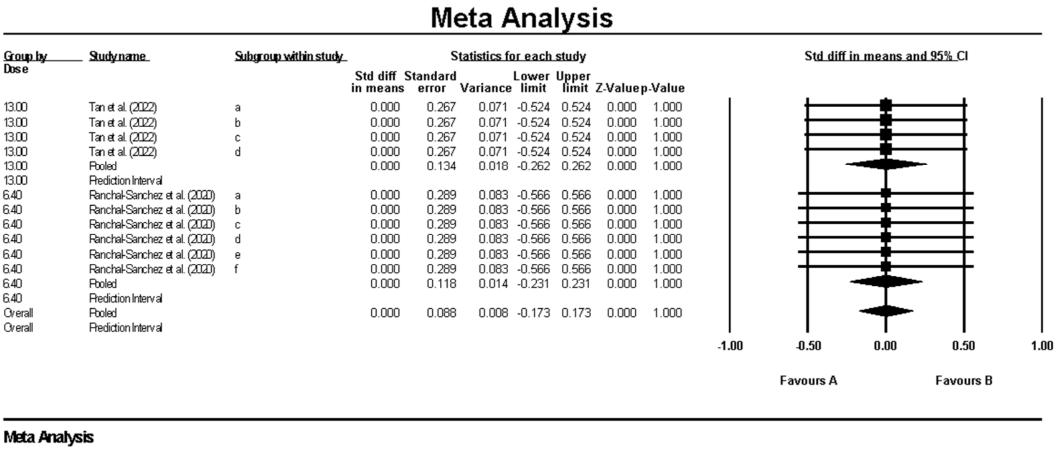

Figure S16. Forest plot demonstrating subgroup analysis by dose for peak velocity (V_{peak}) following placebo (A) and nitrate (B).

Meta Analysis

Group by Exercise	Study name	Subgroup within study	Std diff in means	Standard error	Variance	Lower limit	Upper limit	Z-Value	p-Value
Bench Press	Ranchal-Sanchez et al. (2020)	d	0.000	0.289	0.083	-0.566	0.566	0.000	1.000
Bench Press	Ranchal-Sanchez et al. (2020)	e	0.000	0.289	0.083	-0.566	0.566	0.000	1.000
Bench Press	Ranchal-Sanchez et al. (2020)	f	0.000	0.289	0.083	-0.566	0.566	0.000	1.000
Bench Press	Tan et al. (2022)	c	0.000	0.267	0.071	-0.524	0.524	0.000	1.000
Bench Press	Tan et al. (2022)	d	0.000	0.267	0.071	-0.524	0.524	0.000	1.000
Bench Press	Pooled		0.000	0.125	0.016	-0.245	0.245	0.000	1.000
Bench Press	Prediction Interval								
Squat	Ranchal-Sanchez et al. (2020)	a	0.000	0.289	0.083	-0.566	0.566	0.000	1.000
Squat	Ranchal-Sanchez et al. (2020)	b	0.000	0.289	0.083	-0.566	0.566	0.000	1.000
Squat	Ranchal-Sanchez et al. (2020)	c	0.000	0.289	0.083	-0.566	0.566	0.000	1.000
Squat	Tan et al. (2022)	a	0.000	0.267	0.071	-0.524	0.524	0.000	1.000
Squat	Tan et al. (2022)	b	0.000	0.267	0.071	-0.524	0.524	0.000	1.000
Squat	Pooled		0.000	0.125	0.016	-0.245	0.245	0.000	1.000
Squat	Prediction Interval								
Overall	Pooled		0.000	0.088	0.008	-0.173	0.173	0.000	1.000
Overall	Prediction Interval								

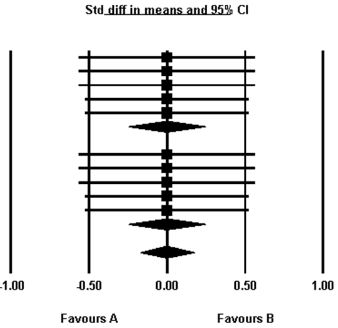

Meta Analysis

Figure S17. Forest plot demonstrating subgroup analysis by exercise modality for peak velocity (V_{peak}) following placebo (A) and nitrate (B).

Meta Analysis

Group by Dose	Study name	Subgroup within study	Std diff in means	Standard error	Variance	Lower limit	Upper limit	Z-Value	p-Value
13.00	Tan et al. (2022)	a	1.000	0.327	0.107	0.358	1.642	3.055	0.002
13.00	Tan et al. (2022)	b	1.000	0.327	0.107	0.358	1.642	3.055	0.002
13.00	Tan et al. (2022)	c	0.000	0.267	0.071	-0.524	0.524	0.000	1.000
13.00	Tan et al. (2022)	d	0.000	0.267	0.071	-0.524	0.524	0.000	1.000
13.00	Pooled		0.473	0.285	0.081	-0.087	1.032	1.656	0.098
13.00	Prediction Interval		0.473			-1.957	2.902		
6.40	Williams et al. (2020)	a	1.000	0.369	0.136	0.276	1.724	2.708	0.007
6.40	Pooled		1.000	0.369	0.136	0.276	1.724	2.708	0.007
6.40	Prediction Interval								
Overall	Pooled		0.670	0.226	0.051	0.227	1.112	2.966	0.003
Overall	Prediction Interval		0.670			-0.996	2.336		

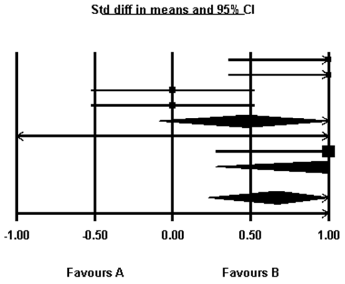

Meta Analysis

Figure S18. Forest plot demonstrating subgroup analysis by dose for mean velocity (V_{mean}) following placebo (A) and nitrate (B).

Meta Analysis

Group by Exercise	Study name	Subgroup within study	Std diff in means	Standard error	Variance	Lower limit	Upper limit	Z-Value	p-Value
Bench Press	Tan et al. (2022)	c	0.000	0.267	0.071	-0.524	0.524	0.000	1.000
Bench Press	Tan et al. (2022)	d	0.000	0.267	0.071	-0.524	0.524	0.000	1.000
Bench Press	Williams et al. (2020)	a	1.000	0.369	0.136	0.276	1.724	2.708	0.007
Bench Press	Pooled		0.282	0.293	0.086	-0.292	0.857	0.963	0.335
Bench Press	Prediction Interval		0.282			-6.117	6.681		
Squat	Tan et al. (2022)	a	1.000	0.327	0.107	0.358	1.642	3.055	0.002
Squat	Tan et al. (2022)	b	1.000	0.327	0.107	0.358	1.642	3.055	0.002
Squat	Pooled		1.000	0.231	0.054	0.546	1.454	4.320	0.000
Squat	Prediction Interval								
Overall	Pooled		0.724	0.182	0.033	0.368	1.080	3.987	0.000
Overall	Prediction Interval		0.724			-0.886	2.334		

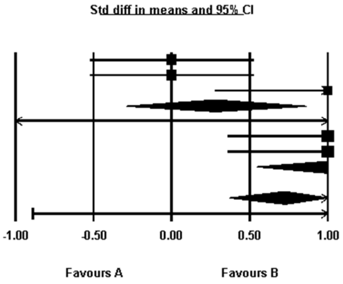

Meta Analysis

Figure S19. Forest plot demonstrating subgroup analysis by exercise modality for mean velocity (V_{mean}) following placebo (A) and nitrate (B).
